# Chiral Flavonoids as Antitumor Agents

**DOI:** 10.3390/ph14121267

**Published:** 2021-12-05

**Authors:** Cláudia Pinto, Honorina Cidade, Madalena Pinto, Maria Elizabeth Tiritan

**Affiliations:** 1 Laboratory of Organic and Pharmaceutical Chemistry, Department of Chemical Sciences, Faculty of Pharmacy, University of Porto, Rua de Jorge Viterbo Ferreira, 228, 4050-313 Porto, Portugal; elsaferreira1971@hotmail.com (C.P.); hcidade@ff.up.pt (H.C.); madalena@ff.up.pt (M.P.); 2 CIIMAR—Interdisciplinary Centre of Marine and Environmental Research, University of Porto, Novo Edifício do Terminal de Cruzeiros do Porto de Leixões, Avenida General Norton de Matos, S/N, 4450-208 Matosinhos, Portugal; 3 CESPU, Institute of Research and Advanced Training in Health Sciences and Technologies (IINFACTS), Rua Central de Gandra, 1317, 4585-116 Gandra, Portugal

**Keywords:** flavonoids, chiral, enantiomers, antitumor, enantioselective synthesis

## Abstract

Flavonoids are a group of natural products with a great structural diversity, widely distributed in plant kingdom. They play an important role in plant growth, development and defense against aggressors. Flavonoids show a huge variety of biological activities such as antioxidant, anti-inflammatory, anti-mutagenic, antimicrobial and antitumor, being able to modulate a large diversity of cellular enzymatic activities. Among natural flavonoids, some classes comprise chiral molecules including flavanones, flavan-3-ols, isoflavanones, and rotenoids, which have one or more stereogenic centers. Interestingly, in some cases, individual compounds of enantiomeric pairs have shown different antitumor activity. In nature, these compounds are mainly biosynthesized as pure enantiomers. Nevertheless, they are often isolated as racemates, being necessary to carry out their chiral separation to perform enantioselectivity studies. Synthetic chiral flavonoids with promising antitumor activity have also been obtained using diverse synthetic approaches. In fact, several new chiral bioactive flavonoids have been synthesized by enantioselective synthesis. Particularly, flavopiridol was the first cyclin-dependent kinase (CDK) inhibitor which entered clinical trials. The chiral pool approaches using amino acid as chiral building blocks have also been reported to achieve small libraries of chrysin derivatives with more potent in vitro growth inhibitory effect than chrysin, reinforcing the importance of the introduction of chiral moieties to improve antitumor activity. In this work, a literature review of natural and synthetic chiral flavonoids with antitumor activity is reported for the first time.

## 1. Introduction

Cancer is a heterogeneous disease resulting from uncontrolled proliferation and deregulation of the cell cycle, producing abnormal cells that are often able to invade and metastasize other organs from the body [1]. According to World Health Organization (WHO), cancer was responsible for about 9.6 million deaths worldwide in 2018, being the second leading cause of death after cardiovascular diseases [2]. In the last year, this global health problem became even more pressing due to the coronavirus disease 2019 (COVID-19) which delayed the diagnosis and treatment due to conditioned access to health care institutions [3]. The growing incidence associated with cancer diseases along with problems of multiple side effects and resistance inherent to classical chemotherapeutic agents renders the search for new compounds with antitumor effects increasingly urgent [4].

Nature is an interesting source of new anticancer drugs [5]. Indeed, some of the drugs currently in therapeutics as anticancer agents are natural products or derived from natural products, most of them being chiral [6]. Flavonoids are a class of natural products with a broad spectrum of pharmacological activities [7]. Many of them are chiral and associated with relevant biological activities, including antitumor by being able to interfere with carcinogenesis processes [8].

## 2. Flavonoids

Flavonoids are important natural products widely present in nature, namely in terrestrial plants and herbs [9], but also in the marine environment [10]. So far, about 8000 different flavonoids have been described that assume a defensive role once they are essentially produced to protect plants from exterior damage including biotic and abiotic factors [11]. With the exposure to stress conditions, such as lack of water or oxygen, extreme temperature or high salinity, the accumulation of these metabolites increases [12]. Among these, temperature is believed to be the most influential factor for the biosynthesis of flavonoids [13]. However, flavonoid biosynthesis is also affected by plant growth regulators such as auxin and cytokinin [12]. Flavonoids can also act as UV filters, signal molecules and on antimicrobial defence as phytoalexins [14].

In plants, many flavonoids are present in flowers and fruits, being responsible for the colour and aroma to attract pollinators that provide seed and spore germination [14].

Chemically, flavonoids are polyphenolic compounds with low molecular weight, characterized by a fifteen carbon skeleton (C6-C3-C6), where two benzene rings (A and B) are linked through a three carbon bridge which usually arises as an oxygenated heterocyclic ring (C) [15].

Depending on the degree of saturation and oxidation of C-ring, and the position of the B-ring, flavonoids can be organized into different classes (Table 1). Flavones, flavonols, flavanones, flavanols, and anthocyanins, include the largest number of compounds in nature, representing the narrow-sense flavonoids with a 2-phenylchromone nucleus [16]. Isoflavonoids are a class of flavonoids with several subgroups, including isoflavones, isoflavanones, isoflavans, pterocarpans and rotenoids, which share a common 3-phenylchromone scaffold where the B-ring is linked to the C-ring in position 3 [17]. Some of these subclasses are made up of chiral compounds which have stereogenic centers [18,19].

Flavonoids are also frequently present in diet, in diverse foods such as vegetables, fruits, tea, wine or cocoa and considered as privileged compounds for cosmetic, medicinal and pharmaceutical applications [20]. The great diversity of structures is reflected in a wide variety of biological activities reported for this class of natural products and their synthetic analogues, namely antitumor, antibacterial, antimicrobial, antiviral, antimalarial, neuroprotective, anti-inflammatory and antioxidant [21]. In recent years, several reports have pointed to the health benefits associated with flavonoids intake, sensitizing the population to the consumption of foods rich in these compounds [22].

Plant polyphenols such as flavonoids are important antioxidants capable of reducing levels of reactive oxygen species (ROS) through chelating transition metals ions such as iron(II)/copper(I) and iron(III)/copper(II), preventing damage and mutation effects on DNA [23]. In addition to their chelating effect, flavonoids also have free radicals scavenging activity, which is dependent on the number and position of hydroxyl groups along with conjugation and resonance effects [24,25]. Dietary flavonoids are also able to bind onto signaling molecules involved in carcinogenesis and regulate their activity.

Because of their structural diversity that allows reaching various biological targets beyond low adverse effects and toxicity, flavonoids are considered a promising class for the discovery of new potential anticancer drugs [26].

However, flavonoids usually have low bioavailability that somehow will affect their biological activity [27]. Consequently, many efforts have been made to improve their bioavailability and absorption through the use of absorption enhancers, delivery systems, alteration of the absorption site and metabolic stability [28]. Moreover, in order to improve the potency and the pharmacokinetic profile of natural chiral flavonoids several synthetic analogues have been prepared as herein reported.

### 2.1. Natural Chiral Flavonoids with Antitumor Activity

**Figure 1:**
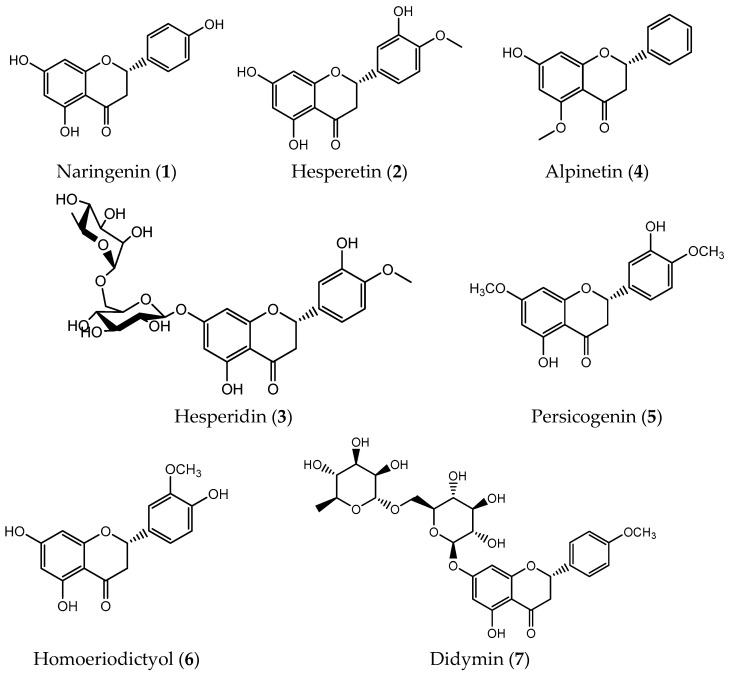
Natural chiral flavanones with antitumor activity.

Flavanones have one stereogenic center in position 2 of the C-ring, making this class of flavonoids chiral molecules (Table 1) [29]. Naringenin (**1**), hesperetin (**2**) and hesperidin (**3**) are found in vegetables and citrus fruits in aglycone forms (Figure 1 [30]. Despite these compounds being able to exist as both enantiomers, in nature they are mainly found as the *S*-enantiomer [31]. Nevertheless, both enantiomers (*R* and *S*) can have biological activities including antioxidant, anti-inflammatory and antitumor [32].

The antitumor effects of naringenin (**1**) have been reported in several studies. Beyond its ability to interfere on the cell cycle, this compound can inhibit the proliferation and migration of hepatocellular carcinoma (HepG2), human gastric cancer (SGC-7901) and human melanoma cells (B16F10 and SK-MEL-28) [33,34,35]. A recent study also suggested the effectiveness of naringenin (**1**) to induce cell apoptosis in A549 lung cancer cells through the activation of caspase-3 cascade [36].

Hesperidin (**3**) and its aglycone hesperetin (**2**) can act on different cancer targets, namely those involved in processes of oxidative stress, inflammation and cell death [37,38]. Their main mechanism of action is related to the induction of apoptosis in many cancer cells such as gastric, breast, prostate, colon, lung and liver [39]. A recent study indicated that hesperetin (**2**) is important to ensure the balance of antioxidant/oxidant state, minimizing the pulmonary toxicity induced by the antineoplastic agent doxorubicin. So, this flavanone could be used as a therapeutic strategy to overcome this problem [38].

Alpinetin (**4**, Figure 1) is a natural flavanone found in seeds of *Alpinia katsumadai* Hayata with anti-inflammatory, antibacterial and antitumor activities [40]. Its antitumor potential is supported by studies that demonstrate that alpinetin (**4**) can inhibit proliferation of lung and gastroenteric cancer cells [40]. Alpinetin (**4**) can also decrease the expression of anti-apoptotic protein Bax while enhancing the expression of pro-apoptotic proteins such as Bcl-2 and caspase-3, translating into inhibition of proliferation and induction of apoptosis on SKOV3 ovarian cancer cells [41]. In addition, this compound is able to block the cell cycle in the G_1_ phase through downregulation of fundamental kinases and suppress the migration of ovarian cancer cells. Alpinetin (**4**) also has the ability to inhibit the proliferation and viability of three pancreatic cancer cells (BxPC-3, PANC-1 and AsPC-1) in a dose- and time-dependent manner [42]. This flavanone is considered as a potential breast cancer chemotherapeutic agent due to its ability to decrease intracellular levels of ROS and the expression of HIF-1α which results in the induction of mitochondria apoptosis and suppression of migration [43].

Persicogenin (**5**, Figure 1) and homoeriodictyol (**6**, Figure 1) are important flavanones isolated from *Rhus retinorrhoea* that present similar structures [44]. Whereas persicogenin (**5**) display anti-mutagenic, antitumor, as well as antibacterial activities, homoeriodictyol (**6**) is relevant to fight osteoporosis and usually used as antioxidant and bitterness masking agent [45,46,47]. According to an MTT assay, these flavanones decrease the survival of MCF-7 (breast cancer), HeLa (cervical cancer), and HT-29 (colon adenocarcinoma) cells through the increased production of ROS and dysfunction of mitochondria. Nevertheless, persicogenin (**5**) induces a more demarcated apoptotic response which is believed to be related to the presence of an additional methyl group on its structure. Interestingly, the selectivity of persicogenin (**5**) is higher for HeLa cells than for MCF-7 whereas homoeriodictyol (**6**) is more selective towards MCF-7 than for HT-29 cells [44].

Didymin (**7**, Figure 1) is a dietary flavonoid glycoside widely present in citrus fruits such as orange, mandarin and bergamot [48]. It behaves as an antioxidant agent whose structure allows scavenging free radicals, attenuating harmful effects associated with products resulting from lipid peroxidation and also activating some antioxidant enzymes [49]. Moreover, recent studies have suggested its antitumor effects mainly on neuroblastoma, lung and breast cancer cell lines although its mechanisms are not completely elucidated [50].

Didymin (**7**) also enhances Raf kinase inhibitory protein (RKIP) and reduces the expression of multiple targets such as cancer-promoting kinases and cyclins, inducing apoptosis and inhibiting proliferation on neuroblastoma cells. In addition, in vivo studies indicate that didymin (**7**) at a dose of 2 mg/kg body weight effectively reduces the tumor size on MYCN-amplified NB xenografts. Gathering all these features, didymin (**7**) is considered a promising compound proposed for neuroblastomas in children [48].

In 2010, Hung et al. studied the antitumor potential of didymin (**7**) both in vitro and in vivo. This flavonoid was shown to induce apoptosis on H460 and A549 lung cancer cells with IC_50_ values of 11.06 µM and 12.57 µM, respectively, through the activation of Fas/Fas ligand apoptosis system. Studies performed with in vivo mice xenograft models revealed that didymin (**7**) at a dose of 6 mg/kg/day notably suppress tumor growth without reported adverse effects [49].

Later, the influence of didymin (**7**) on breast cancer eventually developed by exposure to phthalates was demonstrated. The results suggested that this flavanone (**7**) was able to prevent the progression of this type of cancer, mitigating the processes of invasion, migration and proliferation on MDA-MB-231 cells. The overall results suggested the potential efficacy of didymin (**7**) in preventing phthalate ester-associated cancer aggravation [51].

**Figure 2:**
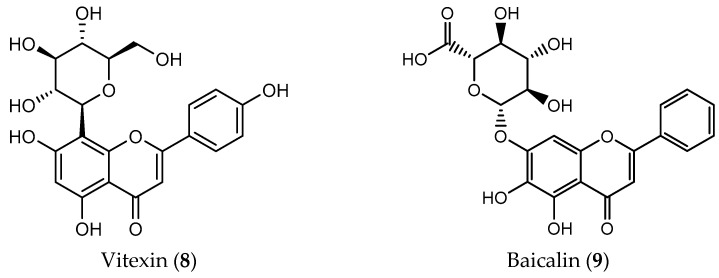
Natural chiral flavones with antitumor activity.

Vitexin (**8**, Figure 2) is a flavone present in many plants such as mung beans, bamboo or passiflora which has many pharmacological activities including antioxidant, anti-inflammatory, antibacterial, antitumor, antiviral and cardioprotective [52]. Beyond demonstrating antitumor efficacy against leukemia, glioblastoma and hepatocellular carcinoma, vitexin (**8**) can induce apoptosis on human non-small cell lung cancer A549 cells through the increased expression of pro-apoptotic protein Bax and suppressing the intracellular pathway PI3K/Akt/mTOR signaling [53]. This compound was also able to induce apoptosis of a drug-resistant colon cancer cell line, suggesting its potential as a chemotherapeutic agent [54].

Baicalin (**9**, Figure 2) is a flavone glycoside abundant on the roots of the traditional Asian herb *Scutellaria baicalensis* that show many biological activities including antioxidant, anti-inflammatory, and antiviral [55]. Many studies point also to its antitumor potential both in vitro and in vivo without marked toxicity [56]. Baicalin (**9**) promoted apoptosis in HT-29 cells in a dose and time-dependent manner as well as inhibition of the tumor growth [55]. Treatment with baicalin (**9**) was responsible for silencing the expression of oncogenic transcription factor c-Myc while decreasing the expression of apoptosis-related oncomiRs involved in cell growth and tumor progression in HT-29, SW-480 (colon adenocarcinoma) and CACO2 cells (colorectal adenocarcinoma) [55]. This flavone can also induce apoptosis in many cancer cell lines such as breast, colon, prostate, lung, gastric, pancreatic, among others, through the control of anti-apoptotic Bcl-2 and pro-apoptotic Bax related to the apoptotic pathway [57]. The expression of caspases 3 and 9 was enhanced on SW1990 (pancreatic adenocarcinoma), A2780 (ovarian cancer) and MG-63 (osteosarcoma) cells treated with flavone **9**. Additionally, **9** increased the expression of tumor suppressor protein p53 on breast cancer cells while on human osteosarcoma cells it enhanced the expression of PARP [58,59]. Furthermore, baicalin (**9**) is also able to interfere with the cell cycle and inhibit the proliferation on many cancer cell lines. For example, treatment with baicalin (**9**) causes G0/G1 cell cycle arrest on U87-MG (glioma), CCRF-CEM (acute lymphocytic leukemia), SKMES-1 (lung), DU145 (prostate), SW620 and HCT116 (colon adenocarcinoma), KIM-1 (hepatocellular carcinoma) and HSC-3 (tongue squamous carcinoma) cells [60,61,62,63,64]. On hepatocellular HepG2 and SMMC-7721 cells, baicalin (**9**) induced G2/M cell cycles arrest [65].

Cytokines and growth factors are involved in the preservation of angiogenesis, which behave as important targets for cancer treatment. On ovarian carcinoma cells, baicalin (**9**) suppress the expression of vascular endothelial growth factor (VEGF), thus reducing cell proliferation [66].

**Figure 3:**
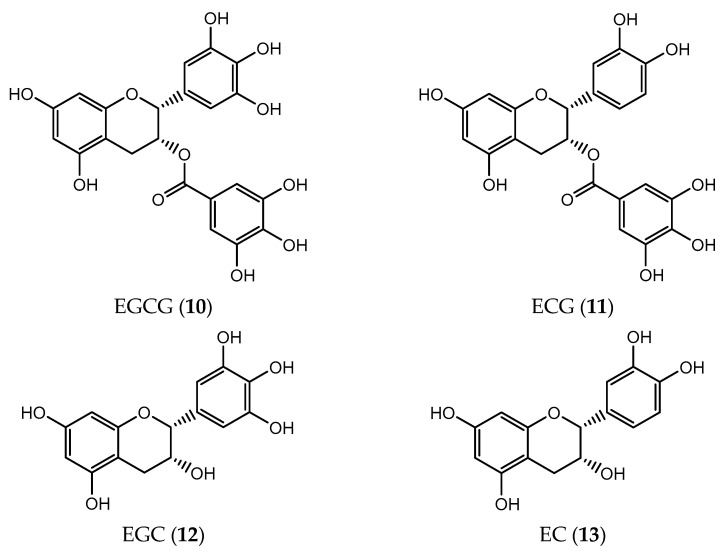
Natural catechins isolated from green tea with antitumor activity.

The major catechins found in green tea, (−)-epigallocatechin-3-gallate (EGCG, **10**), (−)-epicatechin-3-gallate (ECG, **11**), (−)-epigallocatechin (EGC, **12**) and (−)-epicatechin (EC, **13**) showed potent antioxidant activity through their ability to scavenge free radicals and chelate metal ions (Figure 3) [67]. Among them, EGCG (**10**) was reported as the most efficient component to neutralize ROS, contributing to its antitumor effects [68]. Multiple studies reported its potential therapeutic applications on cancer, cardiovascular, liver and neurogenerative diseases and diabetes [69]. EGCG (**10**) main effects as a potential antitumor agent include the inhibition of important targets from the signaling pathways that result in prevention of proliferation and induction of apoptosis on many cancer cell lines including colon, kidney, breast, prostate and brain [70,71]. The use of this flavanol has recently been studied for the treatment of metastatic malignant melanoma once EGCG (**10**) increases apoptosis in IM-9 (myeloma) cells despite its possible hepatic side effects [72,73]. On HT-29 colorectal cell line, treatment with EGCG (**10**) stabilized the activity of transferrin receptor (TfR) and even inhibited the expression of ferritin-H protein [74]. Hepatotoxicity, poor stability and low absorption are some drawbacks inherent to EGCG (**10**) that led to the search for strategies to overcome these limitations, including encapsulation techniques [75]. For example, Chen and co-workers developed an EGCG nanoemulsion that proved to be responsible for activating the AMPK signaling pathway preventing proliferation, migration and invasion on H1299 and A549 lung cancer cells [71].

**Figure 4:**
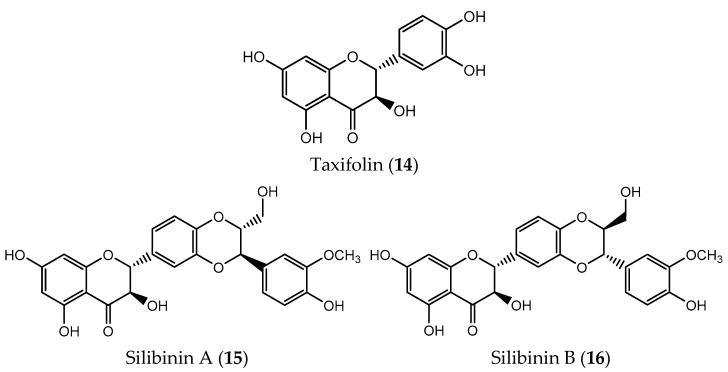
Chemical structures of flavonol taxifolin (**14**) and silibinin diastereoisomers (**15** and **16**).

Taxifolin (**14**, Figure 4) also known as dihydroquercetin, belongs to a flavanol subclass whose major sources include onions, olive oil, grapes, milk thistle and citrus fruits [76]. This flavonoid has many reported pharmacological properties such as antioxidant, anti-inflammatory, antiangiogenic and hepatic-, cardio and neuroprotective effects, its potential in Alzheimer’s disease also being described as it can mitigate the formation of β-amyloid aggregates [77]. Recently, the antitumor activity of flavanol **14** has drawn the attention of several researchers. For example, **14** has been shown to be an efficient inducer of apoptosis and inhibitor of cell growth in both colorectal cancer HCT116 and HT29 cells in a dose dependent manner. The expression levels of cyclin-dependent kinase (CDK) inhibitor p21 and p27 were enhanced on taxifolin (**14**)- treated cells promoting cell cycle arrest in the G2 phase [77]. In addition, **14** was responsible for the induction of apoptosis by the upregulation of pro-apoptotic Bax while downregulating the expression of anti-apoptotic Bcl-2. Finally, more detailed results indicate that taxifolin (**14**) displays a crucial role to change the expression of cell cycle regulators such as β-catenin gene, AKT gene and survivin genes on human colorectal cancer [77]. On another study, researchers reinforced the potential antitumor activity of taxifolin (**14**) through the inhibition of viability, proliferation, migration and invasion of aggressive breast cancer cells both in vitro and in vivo. Again, the downregulation of β-catenin gene demonstrated the taxifolin (**14**) beneficial effects [76].

Cancer stem cells are a recurring problem to fight cancer once they are involved on tumor growth, metastasis, drug resistance and tumor recurrence. Taxifolin (**14**) shows itself to be able to mitigate the viability of A549 and H1975 cells (lung cancer) in a dose-dependent manner by targeting PI3K and TCF4, blocking phosphorylation. Still, A549 cell line proved to be more sensitive to taxifolin (**14**) than H1975 cell line. Likewise, treatment with taxifolin (**14**) suppresses tumor growth in vivo by downregulation of SOX2, OCT2, p-PI3K/PI3K, and TCF4 [78].

In liver carcinoma, taxifolin (**14**) treatment results in decreased expression of matrix metalloproteinases MMP-9 and MMP-2 which translates into inhibition of angiogenesis and invasion [79]. Docking studies showed that the hydroxyl group of taxifolin (**14**) interacts specifically with amino acids from the binding pocket of VEGF, Akt and Hif1α which are upregulated in hepatic cancer cells. The cytotoxic potential of taxifolin (**14**) was proven by the low IC_50_ values in HepG2 (0.15 µM) and Huh7 cells (0.22 µM) [80].

Silibinin or silybin is the major bioactive compound isolated from the milk thistle (*Silybum marianum*) extract, commonly used in the Mediterranean diet. Frequently, an equimolar mixture of two diastereoisomers, Silibinin A (**15**) and Silibinin B (**16**) (Figure 4) is found [81]. This flavonolignan has strong antioxidant and anti-inflammatory activities as a result of the presence of five hydroxyl groups on its structure that contribute to its ability to scavenge free radicals, including reactive nitrogen species (RNS) and ROS [82]. The first reported therapeutic benefits of silibinin were the hepatoprotective effects on acute and chronic liver diseases [83]. However, more studies have drawn attention to its antitumor activity in multiple in vitro and in vivo assays of liver, breast, prostate, skin and colorectal cancers [84,85,86,87]. Silibinin can effectively modulate the expression of many cancer targets and affect signal transduction pathways to promote apoptosis, stop cancer cells proliferation and invasion and mitigate metastasis [88]. In addition, silibinin showed beneficial synergistic action combined with some traditional chemotherapeutic drugs towards ovarian, gastric and hepatocellular cancer cells [89,90,91]. Chemotherapy remains one of the main treatments to fight breast cancer, even though it is increasingly associated with multidrug resistance (MDR) [92]. Silibinin has proven to be able to suppress the growth of MDA-MB-435 and MCF-7 resistant breast cancer cell lines through the inhibition of AKT, ERK and STAT3 pathways, enhancing their susceptibility to the cytotoxic effects of some chemotherapeutic agents [93]. Thereby, many efforts have been made to improve silibinin bioavailability using novel nanotechnological tools in order to allow its clinical use [94].

**Figure 5:**
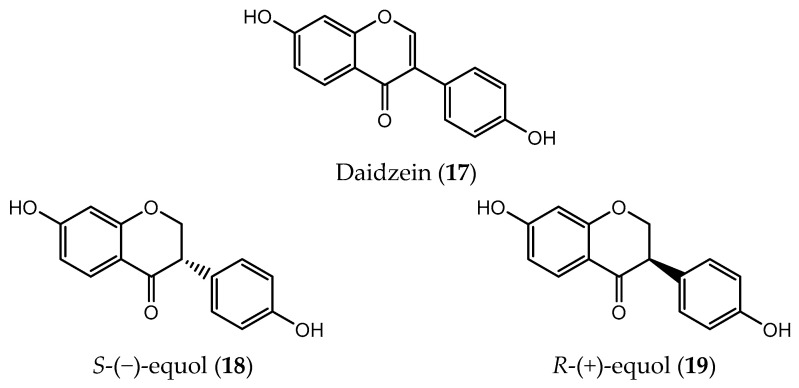
Chemical structure of daidzein (**17**) and its metabolites (**18** and **19**).

Daidzein (**17**, Figure 5), also known as soy isoflavone, through intestinal metabolism form the enantiomer *S*-(−)-equol (**18**) [95]. Isoflavones show potential estrogenic and antiestrogenic effects which is a great concern regarding the consumption of these compounds in patients with breast cancer, since estrogen receptors are overexpressed in tumor cells [96]. Unlike its precursor, *S*-(−)-equol has several structural similarities with estrogen which guarantees a high affinity for estrogen receptor-β (ERβ) [96] while *R*-(+)-equol (**19**) show low affinity for both ERα and ERβ [97]. However, the association between dietary foods containing isoflavones and the risk of developing cancer is not always clear [95]. In the case of prostate cancer, equol acts as an antagonist for dihydrotesterone, promoting the reduction in cell proliferation [98]. In some human breast cancer cells, equol increases estrogenic activity which promotes cell proliferation, but does not affect tumor growth in mice [96]. However, other studies indicate that equol can inhibit the growth and invasion of ERα and ERβ human breast cancer cells through apoptosis induction and cell cycle arrest [99,100].

Because *S*-(−)-equol is the only enantiomer found in the organism as the result of the enantiomeric-specific metabolism of daidzein [101], researchers synthesize both enantiomers in order to study their biological activities [97]. Figure 5 shows the two enantiomers of equol.

Animals that were fed with *R*-(+)-equol showed a significant decrease in the number of palpable tumors contrary to what happen to those who were fed with S-(−)-equol [97].

**Figure 6:**
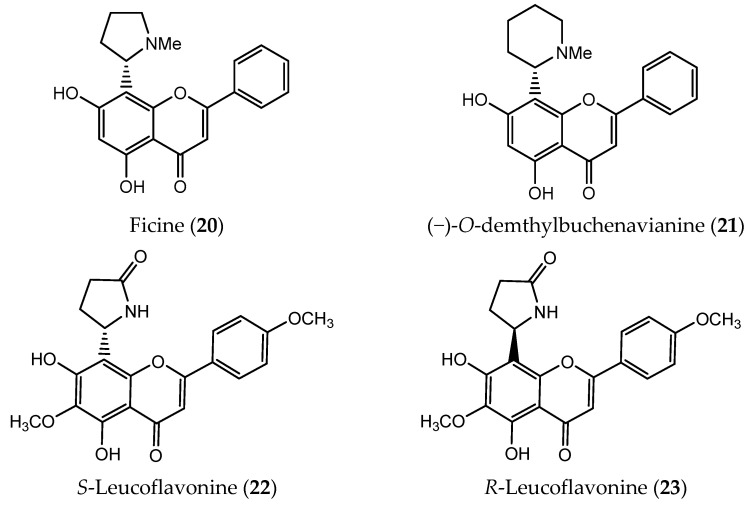
Natural flavoalkaloids with reported antitumor activity.

Flavoalkaloids are relatively rare natural products with a flavonoid framework containing nitrogen fragments attached on position 6 and/or 8 of the A-ring (Figure 6) [102]. In nature, these compounds are found mostly in plants, but also in animals and bacteria [102,103].

In 2012 it was found that natural compounds having a flavoalkaloid skeleton were potent CDK inhibitors, particularly those with *S* configuration and a piperidine or a pyrrolidine group in position 8 [104]. Ficine (**20**, Figure 6), a flavoalkaloid with a pyrrolidine moiety, isolated from *Ficus pantoniana* has proven to be a great inhibitor of CDK1 and CDK5, with an IC_50_ value of 0.04 µM against both isoforms [105].

Another flavoalkaloid that presents a piperidine heterocycle, (−)-*O*-demethylbuchenavianine (**21**, Figure 6), can be found in the fruit of *B. macrophylla*. Although **21** displayed some cytoprotective effects on antiviral assays, this compound was also able to effectively inhibit CDK1 and CDK5 with IC_50_ values of 0.03 and 0.05 µM, respectively [105,106].

*R*- and *S*-Leucoflavonine (**22** and **23**, Figure 6) are flavoalkaloids obtained from *L. canum* leaves, which were isolated as a racemate [103]. After enantioseparation the antitumor activity of both enantiomers was evaluated. While the *S*-enantiomer (**22**) did not show any type of cytoxicity in the cancer cell lines studied (NCI-H1975, PC9, and HepG2), the *R*-enantiomer (**23**) exhibited weak cytotoxicity in HepG2 cells. These results suggest the importance of the stereochemistry for the antitumor activity of leucoflavonine [103].

**Figure 7:**
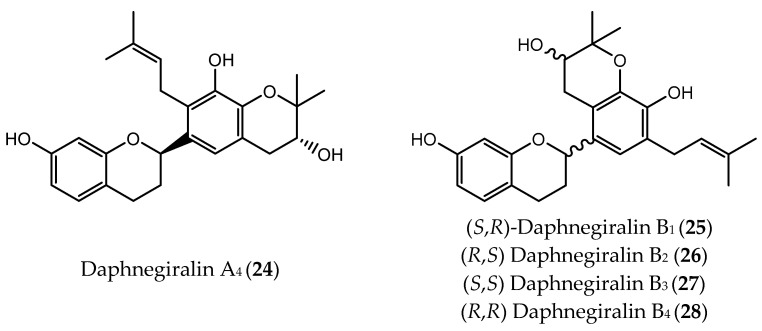
Chemical structure of daphnegiralin A_4_ (**24**) and B_1_–B_4_ (**25–28**) isolated from *Daphne giraldii* with antitumor activity.

In 2016, a group of researchers isolated twelve chiral flavan derivatives from the stem bark and roots of *Daphne giraldii*, including four pairs of enantiomers and two pairs of epimers. The isolation was performed by liquid chromatography (LC) with a Chiralpak AD-H column in reversed elution mode. Daphnegiralin A_4_ (**24**) and daphnegiralins B_1_–B_4_ (**25**–**28**, Figure 7) showed in vitro growth inhibitory activity in liver cancer Hep3B cell line, with IC_50_ values below 10 µM [107].

### 2.2. Synthetic Chiral Flavonoids with Antitumor Activity

There are basically two strategies to achieve enantiomeric pure compounds [108]. One of them refers to the conventional synthesis and further implementation of one resolution method to achieve pure enantiomers from the racemate [108]. This process allows obtaining up to 50% of the desired product, but it is usually time consuming and too expensive when compared to other strategies to achieve enantiomerically pure compounds that may compromise its application on a large scale [109,110]. However, at an early stage of drug discovery and development it is necessary to have both enantiomers to perform biological assays and enantioselective studies in order to understand their pharmacological behavior [111].

Methods describing the enantioseparation of chiral flavonoids are scarce. Enantioseparation of flavonoids by LC with chiral stationary phases (CSP) started with polysaccharide columns, including cellulose and amylose derivatives, followed by cyclodextrin columns [112,113]. Dynamic kinetic resolution has also been recently described [114].

Stereoselective synthesis or asymmetric synthesis is another strategy widely employed to achieve enantiomerically pure compounds having complex structures [115]. Chiral derivatives of flavonoids have been synthesized following multiple approaches.

**Figure 8:**
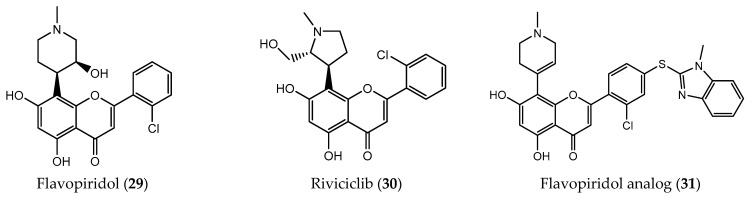
Chemical structure of flavopiridol (**29**) and its analogues (**30** and **31**).

Flavopiridol (**29**, Figure 8) is a semi-synthetic analogue based on the natural bioactive chromone alkaloid, Rohitukine, being the first CDKI to reach clinical trials [116,117]. Flavopiridol competes with ATP to reach the active site of the kinase (CDK1, CDK2, CDK4, CDK6, CDK7 and CDK9), inducing apoptosis by reducing the expression of Bcl-2 anti-apoptotic members, consequently blocking transitions phases G1/S and G2/M from the cell cycle in chronic lymphocytic leukemia and acute myeloid leukemia cells [118]. The chronic and acute leukemia treatment seems to have better responses to the administration of flavopiridol (**29**) in comparison with other chemotherapeutic drugs, namely cytosine, arabinoside or mitoxantrone [119]. In many studies, flavopiridol (**29**) has demonstrated antiproliferative and cytotoxic effects in solid tumors [120]. It was revealed to be a potent CDK7 and CDK9 inhibitor with an IC_50_ value of 10 nM [119,121,122].

Due to its promising results, flavopiridol (**29**) has been the target of several studies, namely, to elucidate its mechanism of action, as well as the possible structural modifications allowing obtaining new CDK inhibitors [105]. Riviciclib (**30**, Figure 8) is a flavopiridol analogue with a pyrrolidine moiety able to induce cell cycle arrest and apoptosis by effectively inhibiting CDK 1 and 9 [123]. It is currently in Phase II clinical trials for the treatment of multiple myeloma, advanced refractory neoplasms and relapsed or refractory mantle cell lymphoma [121,122]. Furthermore, in order to understand the importance of the C-ring for the activity of flavopiridol, several analogues were prepared through the coupling reaction between iodine-flavopiridol with sugars, amino acids and heterocycles using PdG3-Xanthphos precatalyst in mild conditions [118]. Among these, flavopiridol analogues with a benzimidazole group showed greater cytotoxic activity than flavopiridol (**29**). Compound 6-(2-chloro-4-((1-methyl-1*H*-benzo[d]imidazol-2-yl)thio)phenyl)-4-(1-methyl-1,2,3,6-tetrahydropyridin-4-yl)-8-methylene-5,8-dihydronaphthalene-1,3-diol (**31**, Figure 8) showed the best antiproliferative activity, with low IC_50_ values against all tested cell lines, behaving as a CDK9 and GSK3β inhibitor [118].

Chrysin (5,7-dihydroxyflavone) is a dietary phytochemical belonging to the flavone class widely found in honey and propolis which has been shown to have antiproliferative activity in several human tumor cell lines, such as colorectal [124], gastric [125], lung [126], melanoma [127] and prostatic [128]. Moreover, the antitumor activity of this flavone has been demonstrated in hepatocellular [129] and thyroid tumors in animal models [130].

Despite its promising in vitro antitumor activity, chrysin showed disappointing results in in vivo assays due to its low solubility, absorption and rapid metabolism [131].

**Figure 9:**
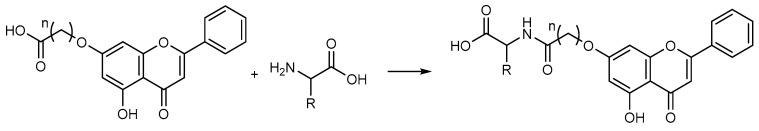
General scheme for synthesis of amino acid derivatives of chrysin.

Amino acids are essential organic chiral small molecules that act as the building blocks of proteins having high biocompatibility [132]. Their introduction in bioactive natural products such as chrysin can be a strategy to improve the interactions and selectivity of these compounds for cancer cells, as well as to improve their bioavailability and minimize adverse effects [133,134]. Therefore, the introduction of amino acids into the synthesis of derivatives of chrysin could lead to an increase in interaction and selectivity to the target-cells, improve the permeability of cell walls and enhance bioavailability [135]. Several approaches can be followed to synthesize amino acid derivatives, such as, for instance, the method of activated esters, which is widely used in peptide chemistry and has been used before to successfully synthesize amino acid derivatives of chrysin (Figure 9) [136].

**Figure 10:**
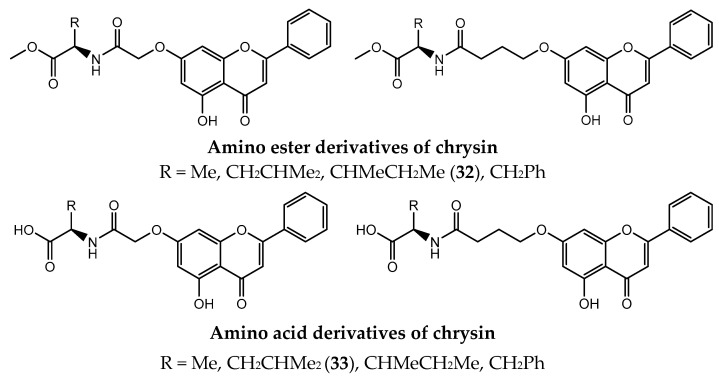
Chemical structure of amino acid derivatives and amino acid ester derivatives of chrysin.

Several derivatives of chrysin were obtained by the incorporation of amino acids alanine, leucine, isoleucine and phenylalanine at C7 position via an acetyl or butyryl group (Figure 10) [131]. Beyond eliminating in vivo problems of chrysin, the introduction of amino acids moiety was considered as a potential pharmacophore responsible for upgrading the antiproliferative activity against cancer cell lines [131]. Among all the synthesized compounds, *N*-[4-(5-hydroxy-4-oxo-2-phenyl-4*H*-chromen-7-yloxy)butyryl]-L-isoleucine methyl ester (**32**) proved to be the most effective and to induce apoptosis on MGC-803 cells (human gastric carcinoma) with an IC_50_ value of 3.8 µM [131].

Later, the same group found out that the derivative *N*-(7-((5-hydroxy-4oxo-2-phenyl-4*H*-chromen-7-yl)oxy)valeryl)-L-leucine (**33**) was shown to induce apoptosis and inhibit the activity of epidermal growth factor receptor (EGFR), a protein tyrosine kinase which is overexpressed in several cancers, beyond having the most potent activity with IC_50_ values of 16.6 μM against MCF-7 cells [134]. Comparing the results of the antitumor activity it was verified that amino acid derivatives have better inhibitory effects than amino acid ester derivatives, possibly due to the formation of a hydrogen interaction between the carboxyl group and the target protein. In addition, chrysin derivatives containing L-leucine showed greater potency by establishing three hydrogen bonds with EGFR, blocking phosphorylation [134].

Quercetin (**34**) belong to the flavonol subclass that is widely found in fruits such as apples, berries, vegetables and teas [137]. The presence of multiple hydroxyl groups in its structure, namely at C3 and a catechol group in B-ring, is responsible for its potent scavenging and metal ions chelate effects [138]. However, beyond the antioxidant activity, this flavonoid has antidiabetic, anti-inflammatory and anti-proliferative activities towards multiple cancer cells including lung, breast, kidney, prostate, colorectal, nasopharyngeal, pancreatic and ovarian [139]. So, due to its biological potential, quercetin can serve as a pharmacophore for drug design strategy in combination with other elements to improve molecular recognition [140].

**Figure 11:**
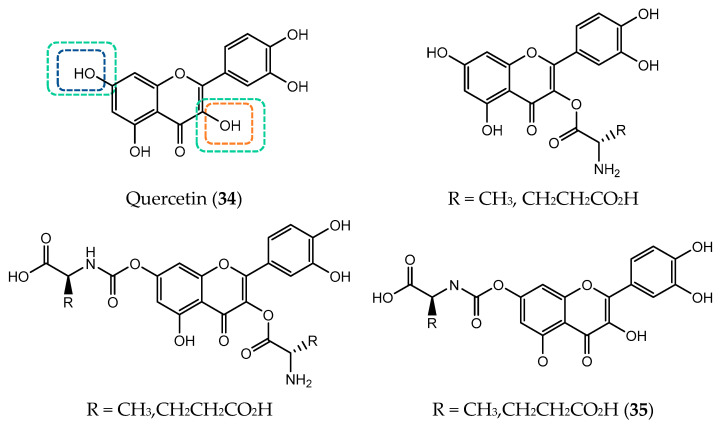
Chemical structure of quercetin (**34**) and quercetin-amino acid conjugates with a carbamate and ester linkage.

The structure of quercetin served as an inspiration for a study based on the development of new MDR modulators. Thus, six new quercetin-amino acid derivatives were prepared by the combination of alanine and glutamic acid at C3 and C7 via carbamate and ester bond (Figure 11) [141]. Compounds with amino acid moiety at the 7 position of quercetin via an amide linkage were the most potent MDR-reversal agents, with IC_50_ values of 0.41 µM (alanine) and 0.14 µM (glutamic acid). Especially, quercetin−glutamic acid conjugate, 7-*O*-Glu-Q (**35**), enhanced about 31 times MDR-reversal activity when compared to quercetin itself, establishing a stronger interaction at the P-glycoprotein (Pgp) binding site. In addition, glutamic acid moiety improved quercetin bioavailability and cellular uptake [141].

**Figure 12:**
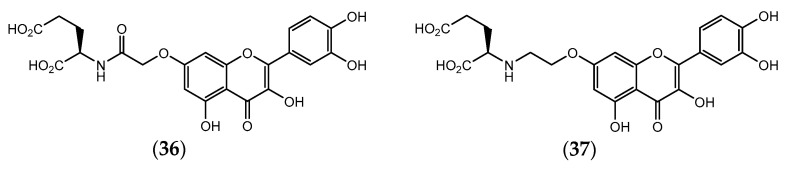
Chemical structure of quercetin- glutamic acid derivatives with non-hydrolysable linkage.

Later, the same research group designed two non-hydrolysable quercetin-glutamic acid derivatives (**36** and **37**, Figure 12) that showed to be more potent MDR-reversal than quercetin (**34**), enhancing the cytotoxicity of tested anticancer drugs such as doxorubicin, vinblastine, paclitaxel and actinomycin D on MES-SA/Dx5 cells, with EC_50_ values ranging between 2.1 and 2.8 µM. Nevertheless, those quercetin-glutamic acid conjugates were not as potent MDR-reversal agents as verapamil [142].

**Figure 13:**
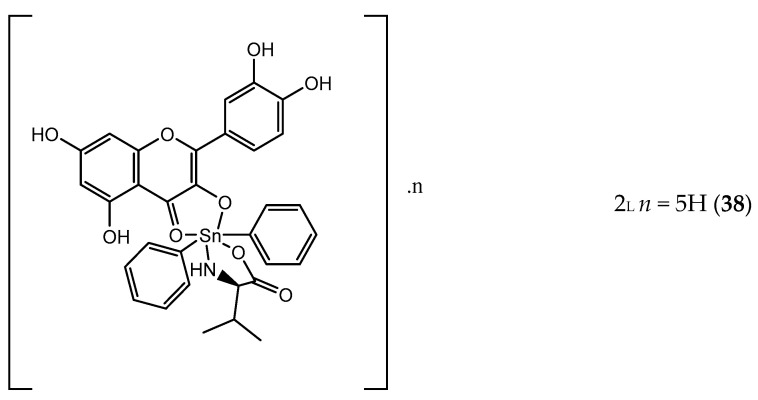
Chemical structure of L-valinequercetin diorganotin (IV) (**38**).

The combination of quercetin (**34**) with metal ions leads to the formation of complexes that, due to their spatial orientation at the active site, allowed obtaining better in vitro and in vivo effects*,* in particular tin and organotin (IV) derivatives [143]. With the addition of organic donor ligands, organotin derivatives can change their coordination number which can go from four to seven [144]. Its functionalization allowed improving biocompatibility, specificity and selectivity as well as reduce toxicity. Organotin (IV) complexes have shown very good IC_50_ values when compared to other drugs in therapeutics [145]. Taking this into account and in order to study the stereoselective recognition with target molecules, valine enantiomer’s was used as a chiral auxiliary to form quercetin organotin derivatives [145]. The conjugation of quercetin pharmacophore with organotin (IV) and with the chiral recognition domain by valine can lead to induce cell death [140]. A complex containing the L-enantiomer was found to have the higher binding affinity with DNA in vitro when compared to D-enantiomer. Among L-enantiomeric complexes, 2_L_ (**38**) was the compound which revealed the best binding ability, showing GI_50_ values below 10 µg/mL, against cervix, breast, hepatoma and pancreas cancer cell lines (Figure 13) [140].

**Figure 14:**
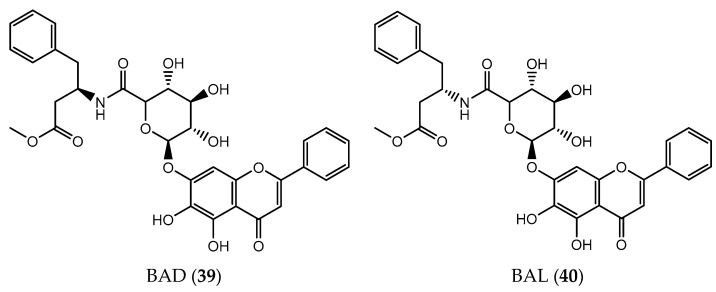
Chemical structure of baicalin derivatives, BAD (**39**) and BAL (**40**).

Recently, data related to molecular docking helped to prepare a pair of chiral baicalin (**9**) derivatives, BAD (**39**) and BAL (**40**) through the conjugation with phenylalanine methyl ester moiety in order to improve the antitumor activity of this bioactive compound (Figure 14). the introduction of phenylalanine methyl ester is a well-known strategy to confer chirality that will give spatial specificity and enhance the selectivity of cancer cells [146]. Results show that BAD (**39**) and BAL (**40**) have better antitumor activity than baicalin itself against MCF-7, T47D (human breast cancer), H460 and A549 cells (lung cancer). Particularly on lung cancer cell line A549, BAL (**40**) showed better affinity, followed by BAD (**39**) and by baicalin (**9**). However, BAD (**39**) may lead to a higher inhibition rate over time since usually D-enantiomer of chiral drugs have better access to cancer cells [147].

**Figure 15:**
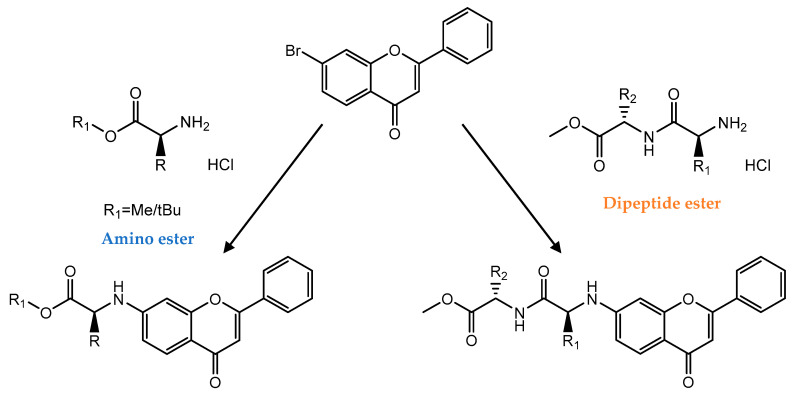
General scheme for synthesis of flavone amino acid derivatives through the Buchwald-Hartwig reaction.

The development of Buchwald-Hartwig amination of different bromoflavones with amino acid and peptide derivatives as nitrogen source giving unique structures allowed the synthesis of a series of flavone amino acid derivatives (Figure 15) [117]. The synthesis of these unique derivatives was also demonstrated by the deprotection of flavone-amino acid hybrids followed by classical peptide synthesis. The biological assays exhibited that these special structures are promising cytotoxic compounds against different cell lines. The previously observed racemization, which occurred during the synthesis of flavone-amino acid hybrids, was successfully prevented in most cases [117].

**Figure 16:**
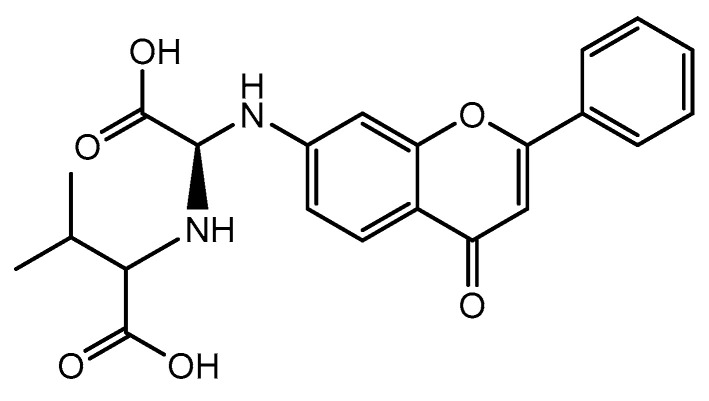
Chemical structure of flavone−dipeptide hybrid L-Val-OH (**41**).

It is important to refer that the coupling enantiomerically pure amino acid under basic conditions usually leads to racemization. In addition, the racemization process can also occur in reactions with palladium catalyst, but this situation can be mitigated by combining bulky ligands such as BINAP with catalysts that form a more stable complex during reaction [148]. In addition, high temperature and long reaction times lower the enantiomeric ratio although suggesting good overall yields. All flavone dipeptide hybrids that were studied show cytotoxic effects on different cancer cell lines such as osteosarcoma, leukemia, lung, and colorectal cancer [117]. Among them, flavone−dipeptide hybrid constituted by L-Val-OH (**41**) moiety shows the lowest IC_50_ value (9.2 µM) against T-lymphoblastic leukemia cell line (Figure 16) [117].

**Figure 17:**
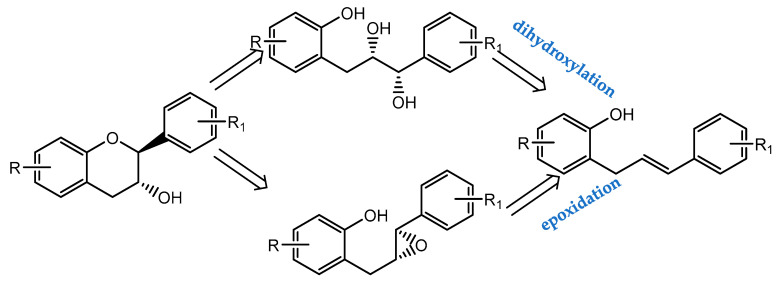
Dihydroxylation and epoxidation to construct chiral centers (Retrosynthesis).

Total synthesis of chiral flavanols has been gaining importance over the years giving rise to new compounds with unique structure and promising pharmacological activities. The way in which stereogenic centers are built and stereoselective cyclization occurs to form the C ring can be achieved through Sharpless asymmetric dihydroxylation, Shi epoxidation, Sharpless epoxidation and chiral resolution (Figure 17) [149].

**Figure 18:**
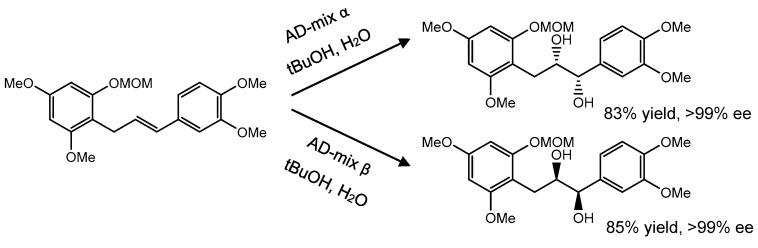
Sharpless asymmetric dihydroxylation to achieve chiral centers.

Asymmetric dihydroxylation of olefins with AD-mix-α or AD-mix-β catalyst create *syn*-diols with good yields and optical purity (Figure 18) [150]. In this way, two stereogenic centers were obtained in a single reaction step that make this reaction very effective. However, the hydroxyl of the phenol group from the starting reagent needed to be protected with groups such as methoxymethyl (MOM), *t*-butyldimethylsilyl or benzyl (Bn) before asymmetric dihydroxylation [151]. Then, the chiral diols by cyclization and deprotection formed the respective flavanols [149].

Flavanols cyclization can occur by different approaches in order to obtain several compounds. The first reported method involved the removal of the protecting group at position 2′ followed by ring formation and thereafter acetylation in mildly acid conditions to generate 2,3-*cis* and 2,3-*trans*-flavanols derivatives with moderate yields and high enantiomeric excess, with the optical integrity not affected. However, this method lacks from low selectivity for *cis/trans* configurations [152].

In 2001, a research group managed to proceed with the synthesis of EGCG (**10**) through stereospecific cyclization of the Sharpless asymmetric dihydroxylation product. The first reaction steps were similar to those previously mentioned, but this time the diol reacted with triethyl orthoformate in the presence of pyridinium *p*-toluenesulfonate to give an *ortho* ester that would quickly form the ring, giving rise to the *trans* product. The desired *cis* product was obtained through *trans*-intermediate oxidation followed by ketone reduction with L-selectride. Lastly, esterification with Pd(OH)_2_ catalyst allowed the enantioselective synthesis of EGCG (**10**) despite low overall yield [151].

In 2005, analogues of ECG (**11**) with B-ring modifications were obtained through an alternative route of C-ring cyclization. Instead, the cyclic orthoformate intermediate treated with acetyl bromide led to a ring opening intermediate. The subsequent addition of K_2_CO_3_ was responsible for cyclization and deformylation to give *cis*-2,3 product. The syntheses were concluded by DCC-induced coupling with 3,4,5-tri(benzyloxy)benzoic acid and debenzylation with Pd(OH)_2_ addition to result in the enantiomeric pure flavanols [153].

**Figure 19:**
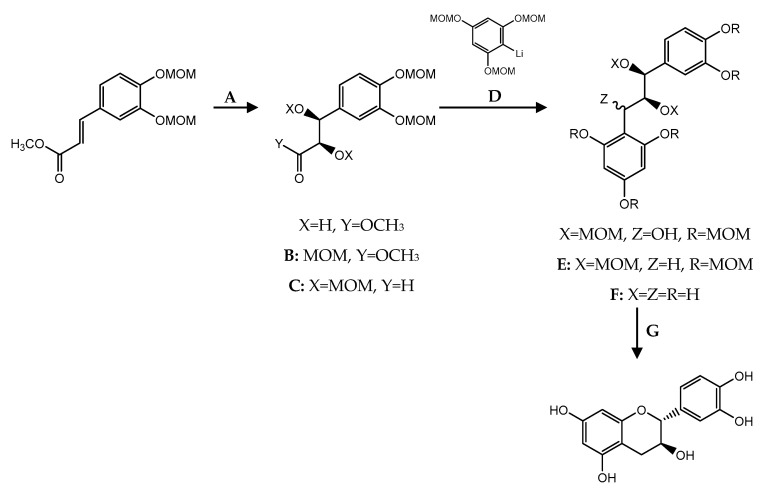
Cyclization by intramolecular Mitsunobu reaction. (**A**) AD-mix α, MeSO_2_NH_2_, *t*BuOH, H_2_O, 100%; (**B**) MOMCl, *i*-Pr_2_NET, CH_2_Cl_2_, 89%; (**C**) DIBAL-H, PhMe, −78 °C, 87%; (**D**) *n*-BuLi, THF, −78 °C, 53%; (**E**) (i) NaH, imidazole, CS_2_, CH_3_I, THF, 99%, (ii) *n*-Bu_3_SnH, AIBN, benzene, 80 °C, 91%; (**F**) 2% HCl, MeOH, 50 °C, 49%; (**G**) PPh_3_, DEAD, THF, 50%.

A method developed in 2002 with catalyst AD-mix-α and methane sulfonamide used on *trans*-methyl cinnamate derivative led to (2*R*,3*S*)-dihydroxyester diastereomer with high enantiomeric excess (>99%) (Figure 19). To avoid epimerization, intramolecular Mitsunobu reaction using triphenylphosphine (PPh_3_) and diethyl azodicarboxylate as catalyst ensured the formation of flavanol with moderate yield. Later, from the respective diols, four flavanols with different substitution patterns and electron densities were synthesized by Mitsunobu reaction in only one step [149].

In 2006, a new cyclization method via halogen-metal exchange was developed [154]. This method started with an asymmetric dihydroxylation by AD-mix-β to achieve a triol. Then, sulfonylation followed by K_2_CO_3_ addition to form an epoxide that by Mitsunobu reaction with substituted phenol formed an oxirane group. The oxirane group ended up being cleaved resulting in an alcohol group that needed to be protected to obtain bromide. This intermediate together with Ph_3_MgLi and HMPA provide the desired product. The last step corresponds to protecting groups release.

Aryl epoxides cyclization of many catechins derivatives was achieved with good yields by AuCl_3_/AgOTf/PPH_3_ mixture under mild conditions [155]. This cyclization method allowed obtaining a large library of this family of compounds with good yields.

In 2010 a series of epi-catechins were synthesized under stereospecific controlled conditions based on an intramolecular nucleophilic substitution followed by the sulfynil-metal exchange and finally cyclization. The nucleophilic substitution reaction was slowly carried out giving the respective product as a mixture of diastereomers. Then, Li_2_NiBr_4_ was added to remove the epoxide ring while triethylsilyl (TES) triflate gave diastereomeric bromides that were obtained after cyclization [156].

Two years later, a new synthetic method was developed in order to achieve polyphenols from the catechin class through the reaction of regioselective lithiation of fluorobenzene and enantiomeric epoxy alcohol by BF_3_.OEt_2_. Before pyran cyclization under basic conditions, the hydroxyl group was protected at the same time that the silyl group was cleaved in order to give the *cis* product with good yields [157].

**Figure 20:**
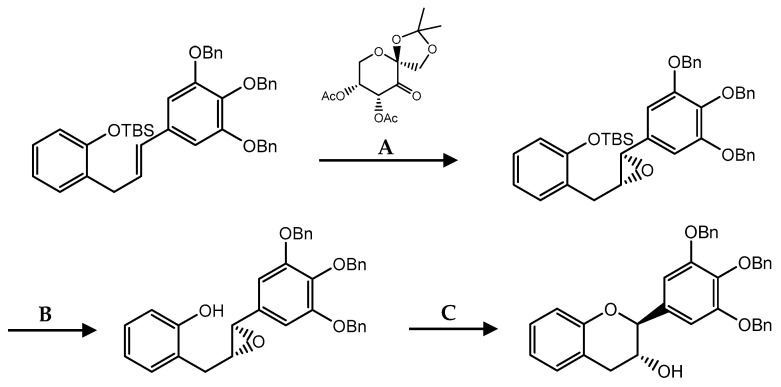
Flavanols synthesis by Shi’s asymmetric epoxidation. (**A**) Oxone, MeCN, DMM, phosphorus buffer; (**B**) TBAF, AcOH, THF; (**C**) CSA, CH_2_Cl_2_.

Another method to construct stereogenic centers corresponds to Shi asymmetric epoxidation (Figure 20) [158]. Taking advantage of the *E*-double bound with fructose derivative addition, oxone and phosphorus buffer in a solvent mixture led to the enantioselective formation of epoxide with good yields and high enantiomeric excess. Then, tetra-n-butylammonium fluoride (TBAF) was added to remove the silyl ether protecting group (TBS) thus allowing the *endo*-cyclization reaction with camphorsulfonic acid (CSA) treatment [159].

**Figure 21:**
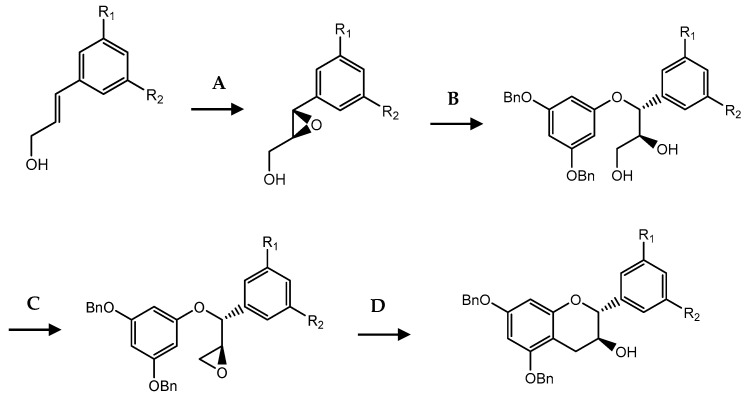
Flavanols synthesis by Sharpless epoxidation. (**A**) Diethyl-L-tartrate, Ti(Por)_4_, *t*-BuOOH; (**B**) 3,5-Dibenzoxyphenol, NaH, THF, H_2_O; (**C**) Pyridine, *p*-tosyl chloridre, r.t, 2 d then K_2_CO_3_; (**D**) HFIP, reflux. Adapted from [149].

More recently, based on Sharpless asymmetric epoxidation, the synthesis of (−)-epicatechin gallate (ECG) (**11**) analogues was improved (Figure 21) [160]. The formation of enantiomeric pure epoxides with excellent enantiomeric excess was achieved from cinnamoyl alcohol reaction with diethyl-L-tartrate, titanium isopropoxide and *t*-butyl hydroperoxide in dichloromethane. The epoxide was then treated with 3,5-dibenzoxyphenol to give diols. Through reaction heating and the addition of hexafluoro-2-propanol (HFIP) they obtained catechin in good quantities, despite the fact that they could be improved by extending the reaction time. The desired epicatechin stereochemistry was achieved by oxidation with Dess–Martin periodinane followed by reduction with L-selectride [149].

Thioflavonoids are synthetic analogues of flavonoids with a sulfur-containing heterocycle [161,162]. It is recognized that these compounds have several biological activities such as antitumor, antidepressive, anti-inflammatory, antiarthritic and antimicrobial, some of them being more potent than flavonoids [163]. Given their abilities, thioflavonoids drive the interest of scientists in synthesis, bioactivity and optical materials fields [162].

Thioflavonoids can be synthesized from the alkynylation of thiochromones [164]. However, thiochromones are less reactive compounds due to the presence of a sulfur electron lone pair which is responsible for a high degree of delocalization, consequently increasing the electronic density in the system [162].

**Figure 22:**
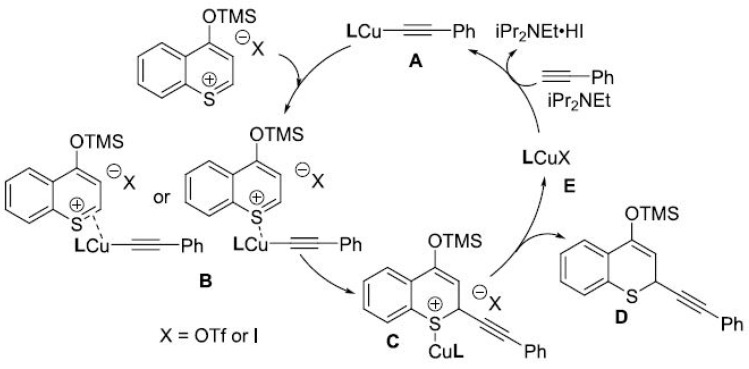
Mechanism of reaction of thioflavonoids as reported by Meng et al. [162].

The first studies started with unsubstituted thiochromone and phenylacetylene as substrates and CuI as the catalyst but whose product stereoselectivity was not properly controlled. Therefore, several chiral ligands have been studied in order to obtain good enantiomeric excess and yields [162]. Phosphoramidite ligands, especially with the a more hindered pentaflourophenyl group led to the better yields (95%) as well as the highest enantioselectivity (92%) [162]. Then, the reaction was studied for substituted thiochromones in position 2, in order to understand the extent of the substrate. It was found that the electronic properties from the substituents did not significantly alter the reaction´s reactivity and stereoselectivity [162]. Basically, the reaction started with the formation of copper acetylide with a base. In its turn, the addition of trimethylsilyl trifluoromethanesulfonate (TMSOTf) activated the thiochromone to form 4-((trimethylsilyl)-oxy)thiochromenylium that can coordinate to copper acetylide by the stabilization from the counteranion. Then, acetylide goes to migratory insertion, forming another complex which, after that, can release the silyl enol ether getting the final product (Figure 22) [162].

Natural and synthetic chiral flavonoids presented in this review with the most promising antitumor activity are summarized in Table 2.

## 3. Conclusions

Flavonoids are natural products with diversified structure widely produced by plants and fruits that demonstrate several biological activities, the antitumor activity being one of the most reported. Among natural flavonoids with antitumor effect, some classes such as flavanones, flavanols, and isoflavanones are chiral, showing one or two stereogenic centers in the flavonoid nucleus namely in positions 2 or 3 of the C-ring. In addition to these classes of natural flavonoids, some chiral flavonoids with in vitro growth inhibitory activity showed the presence of stereogenic centers at the side chains, namely flavonoid glycosides and flavoalkaloids.

Considering synthetic chiral flavonoids, flavopiridol (**29**) is a CDK inhibitor that inspired many researchers to search for new analogues with antitumor activity through structural modifications. Among flavopiridol analogues, those with a benzimidazole group showed greater cytotoxic activity than flavopiridol (**29**).

Most of the activities presented by flavonoids include the inhibition of some enzymes. It is known that the active sites of enzymes are characterized by a highly stereoselectivity. Therefore, the flavonoid interaction with the chiral macromolecule can be modulated by the absolute configuration that leads to different pharmacodynamic effects since one of the enantiomers can have a greater affinity and potency than the other. From these characteristics it can be inferred that to better understand their pharmacological effects, it is necessary to have both enantiomers in enantiomerically pure form.

The synthesis of chiral flavonoids through the incorporation with amino acids has been reported as an effective strategy to improve the antitumor activity of natural flavonoids. For example, to overcome the drawback of low bioavailability of chrysin, baicalin (**9**) and quercetin (**34**), these natural flavonoids were used as substrates to obtain new chiral derivatives with antitumor activity using this synthetic strategy. Despite the association of these flavonoids with amino acids resulting in the improvement of the antitumor activity, the evaluation of both separated enantiomers was not explored. Therefore, in the future it will be important to obtain new libraries of chiral flavonoids with enantiomeric pairs in order to conduct enantioselectivity studies.

Stereoselective synthesis allows the construction of molecules with complex structures from achiral precursors through rational stereoselective synthetic transformations under controlled conditions. In the last years, asymmetric synthesis has been widely used in order to improve the synthesis of natural bioactive compounds available for drug discovery and development. However, the search for better approaches for the synthesis of both enantiomers of chiral flavonoids is still necessary in order to explore the enantioselectivity in antitumor activity.

## Figures and Tables

**Table 1:** Basic skeleton structure of classes of flavonoids.

C ring	Saturated	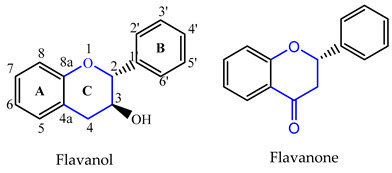
	Insaturated	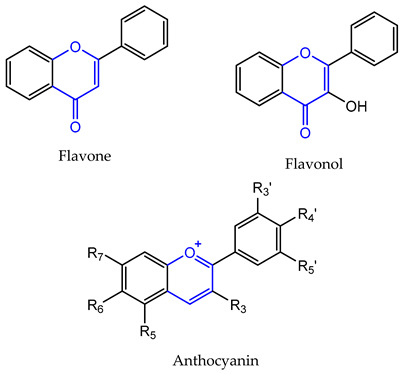
B ring	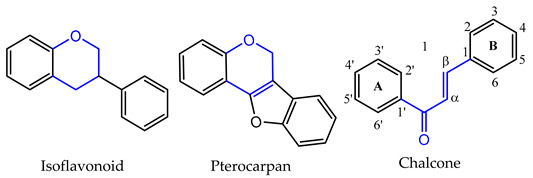	

**Table 2:** Chiral flavonoids with antitumor activity.

Flavonoid Subclass	Name	Cancer Cells/Effects	Ref.
Flavanone	Naringenin (**1**)	hepatocellular carcinoma (IC_50_ = 100 µM) gastric cancer (IC_50_ = 10 µM) melanoma (IC_50_ = 3 µM) non-small-cell lung carcinoma (IC_50_ = 100 µM)	[35,165]
	Hesperetin (**2**)	gastric (IC_50_ = 40 μM) breast (IC_50_ = 20 μM) prostate (IC_50_ = 90 μM) colon (IC_50_ = 100 μM) lung (IC_50_ = 40 μM) liver (IC_50_ = 87 μM)	[39]
	Alpinetin (**4**)	lung (IC_50_ = 25 μM) gastric (IC_50_ = 120 μM) ovarian (IC_50_ = 50 μM) pancreatic (IC_50_ = 60 μg/mL)	[40,41,42,166]
	Persicogenin (**5**)	human cervical cancer (IC_50_ = 500 μg/mL) breast carcinoma (IC_50_ = 500 μg/mL) human colon cancer (IC_50_ = 500 μg/mL)	[44]
	Homoeriodictyol (**6**)	human cervical cancer (IC_50_ = 500 μg/mL) breast carcinoma (IC_50_ = 500 μg/mL) human colon cancer (IC_50_ = 250 μg/mL)	[44]
	Didymin (**7**)	neuroblastoma (IC_50_ = 50 μM) lung (IC_50_ = 11.06 μM)	[50,52,167]
Flavone	Vitexin (**8**)	leukemia (IC_50_ = 200 μM) glioblastoma (IC_50_ = 32 μM) hepatocellular carcinoma (IC_50_ = 5 μM) lung carcinoma (IC_50_ = 40 μM)	[53,168,169,170]
	Baicalin (**9**)	breast (IC_50_ = 100 μM) colon (IC_50_ = 20 μM) prostate (IC_50_ = 150 μM) lung (IC_50_ = 80 μg/mL) gastric (IC_50_ = 80 μM) osteosarcoma (IC_50_ = 25 μM)	[60,167,171,172,173,174]
	Ficine (**20**)	CDK1 and CDK5 inhibition (IC_50_ = 0.04 µM)	[105]
	(−)-*O*-demthylbuchenavianine (**21**)	CDK1 inhibition (IC_50_ = 0.03 µM) CDK5 inhibition (IC_50_ = 0.05 µM)	[105]
	*R*-Leucoflavonine (**23**)	hepatocellular carcinoma (IC_50_ = 52.9 µM)	[103]
	Flavopiridol (**29**)	CDK1 inhibition (IC_50_ = 30 nM) CDK7 inhibition (IC_50_ = 10 nM) CDK9 inhibition (IC_50_ = 3 nM) colon-carcinoma (IC_50_ = 20 nM) breast cancer (IC_50_ = 75 nM) gastric adenocarcinoma (111 nM)	[175,176,177]
	Riviciclib (**30**)	CDK1 inhibition (IC_50_ = 79 nM) CDK9 inhibition (IC_50_ = 20 nM)	[177]
	**32**	gastric carcinoma (IC_50_ = 3.8 µM)	[131]
	**33**	breast (IC_50_ = 16.6 μM)	[134]
	Flavone−dipeptide hybrid L-Val-OH (**41**)	leukemia (IC_50_ = 9.2 µM)	[117]
Flavonol	Taxifolin (**14**)	colorectal (IC_50_ = 40 µM) breast (IC_50_ = 10 µM) lung (IC_50_ = 25 µM) skin (IC_50_ = 80 µM)	[178]
	Quercetin−glutamic acid conjugate 7-O-Glu-Q (**35**)	MDR uterine sarcoma (IC_50_ = 0.14 μM)	[141]
	L-valinequercetin diorganotin(IV) (**38**)	cervix (GI_50_ < 10 μg/mL) breast (GI_50_ < 10 μg/mL) liver (GI_50_ < 10 μg/mL) pancreatic (GI_50_ < 10 μg/mL)	[140]
Flavanol	(−)-epigallocatechin-3-gallate (EGCG) (**10**)	prostatic adenocarcinoma (IC_50_ = 39 µM) colon (IC_50_ = 3 µM) adrenal (IC_50_ = 20 µM) breast (IC_50_ = 20 µM) melanoma (IC_50_ = 7 µM) pancreatic (IC_50_ < 50 µM)	[179,180,181,182]
	Daphnegiralin A_4_ (**24**)	hepatocellular carcinoma (IC_50_ = 5.1 µM)	[107]
	Daphnegiralins B_1_: (2-*S*,2′-*R*) (**25**) Daphnegiralins B_2_: (2-*R*,2′-*S*) (**26**)	hepatocellular carcinoma (IC_50_ = 6.1 µM)	[107]
	Daphnegiralins B_3_: (2-*S*,2′-*S*) (**27**) Daphnegiralins B_4_: (2-*R*,2′-*R*) (**28**)	hepatocellular carcinoma (IC_50_ = 5.4 µM)	[107]
Isoflavone	*S*-(−)-equol (**18**)	breast cancer (IC_50_ = 10 µM) prostate cancer (IC_50_ = 5 µM)	[97,183]
